# Vaginal cuff dehiscence following transvaginal oocyte retrieval: a case report

**DOI:** 10.1186/s40738-020-00085-0

**Published:** 2020-09-02

**Authors:** Sarah K. O’Connor, David A. Ryley, Charles W. Obasiolu, Katharine M. Esselen, Christine C. Skiadas, Wendy Kuohung

**Affiliations:** 1 Department of Obstetrics & Gynecology, Boston Medical Center, Boston, MA USA; 2 Harvard Medical School, Boston IVF, Waltham, MA USA; 3 Harvard Medical School, Atrius Health, Boston, MA USA; 4 Division of Gynecologic Oncology, Beth Israel Lahey Health Boston, Boston, MA USA

**Keywords:** Vaginal cuff dehiscence, Infertility, Vaginal oocyte retrieval, Endometrial cancer

## Abstract

**Background:**

Vaginal cuff dehiscence (VCD) is a rare but potentially serious complication following hysterectomy with an estimated incidence of 0.14–1.4%. There is a wide range of risk factors thought to contribute to VCD, but due to its rare occurrence, much still remains to be learned about the true impact of risk factors leading to dehiscence. We present here the second known report of VCD to occur in a patient undergoing transvaginal oocyte retrieval during her fertility treatment. This case highlights what may become a more common clinical scenario as more premenopausal women are diagnosed with reproductive tract cancers and access assisted reproductive therapies to preserve fertility.

**Case presentation:**

Our patient is a 35-year-old G1 P0 A1 who had undergone ovary-sparing total laparoscopic hysterectomy (TLH) following diagnosis of endometrial adenocarcinoma. She underwent two in-vitro fertilization (IVF) cycles after TLH to bank frozen blastocysts, the first vaginal oocyte retrieval (VOR) taking place 12 weeks following hysterectomy. She experienced VCD during her second VOR that occurred 17 weeks after TLH, the second case of VCD to be reported in the literature during fertility preservation treatment following hysterectomy. The patient underwent an emergent and uncomplicated repair of the defect vaginally the same day.

**Conclusions:**

Currently there are no guidelines in place for women who have undergone hysterectomy with regard to when they can begin fertility treatment in the post-operative period. Based on now two case reports, it is worth considering extension of the typical 6-week timeline of avoidance of vaginal procedures to allow for full cuff healing. Infertility providers should also be mindful of limiting transvaginal ultrasounds where possible to reduce force along the cuff.

## Background

Vaginal cuff dehiscence (VCD) is a rare but potentially serious post-operative complication of hysterectomy with an estimated incidence of 0.14 to 1.4% [1]. VCD with evisceration occurs in the majority of reported cases with an estimated incidence of 0.032 to 1.2% [1]. VCD can present with a range of symptoms including abdominal and pelvic pain or pressure, leaking of vaginal fluid, vaginal bleeding, or sometimes no symptoms with identification on clinical examination [2]. Identification of VCD requires immediate surgical management to prevent additional complications including bowel prolapse/necrosis and sepsis. There is a wide range of risk factors thought to contribute to the development of VCD [1, 3]. As originally outlined by Nezhat et al., these risk factors can be categorized as those that interrupt healthy wound healing (malignancy, diabetes, local infection, etc), increase intra-abdominal pressure (gynecologic intervention, sexual activity, chronic cough or constipation, etc), or are related to surgical methods (mode of hysterectomy with higher dehiscence rates seen for minimally invasive cases, cuff closure techniques, etc) [3]. In case reports, clear precipitating factors are often cited as those causing the dehiscence, but more often the source cannot be clearly identified [2, 4]. It is likely that VCD is multifactorial in etiology. In this report we describe a patient who experienced VCD diagnosed after oocyte retrieval 17 weeks status post total laparoscopic hysterectomy for endometrial cancer.

## Case presentation

The patient was a 35-year-old G1 P0 A1 with a past medical and surgical history notable for asthma, subclinical hypothyroidism on levothyroxine, and prior laparoscopic ovarian cyst removal who originally presented to care for infertility of 2 years’ duration. She had had one biochemical pregnancy at age 32. Her body mass index was 20.5. Laboratory evaluation was notable for a borderline day 3 FSH of 9.14 mIU/ml, and AMH was low at 0.15 mg/ml. Semen analysis was normal. During her evaluation, she underwent a hysteroscopic polypectomy with pathology that demonstrated endometrial adenocarcinoma. In an attempt to perform fertility-sparing treatment for presumed stage IA grade 1 endometrioid cancer and postpone hysterectomy, the patient was treated with progestin therapy including levonorgestrel intrauterine device (IUD) placement and megestrol acetate in conjunction with repeat endometrial biopsies every 3 months as surveillance for one and a half years. Unfortunately, her biopsies continued to demonstrate persistent low-grade endometrial adenocarcinoma, and the decision was made to perform hysterectomy with ovarian conservation and post-operative oocyte collection. The patient underwent an uncomplicated total laparoscopic hysterectomy, bilateral salpingectomy, and bilateral sentinel lymph node dissection with final pathology demonstrating stage IA, grade 1 endometrioid endometrial carcinoma. At the time of surgery, colpotomy was carried out with L-hook monopolar cautery and closure of the cuff with a running V-loc suture in two layers. Her gynecologic oncologist approved IVF treatment starting 8 weeks after hysterectomy after a normal 4-week post-operative exam.

The patient underwent two antagonist IVF cycles with plans for PGT-A. During her first cycle that began 11 weeks after TLH, she received 450 units of FSH (Gonal F®, EMD Serono, Rockland, MA, USA) and 150 units of human menopausal gonadotropin (Menopur®, Ferring Pharmaceuticals, Parsippany, NJ, USA) daily; 0.25 mg of cetrorelix acetate (Cetrotide®, GnRH antagonist EMD Serono, Rockland, MA, USA) was started on day 5. Transvaginal ultrasound scans and labwork were performed every 1–3 days during controlled ovarian stimulation until day 10 of stimulation, when peak estradiol was 1081 pg/mL and 5 follicles greater than 11 mm were seen (the largest measuring 22.9 mm). Oocyte maturation was triggered with subcutaneous choriogonadotropin alfa injection, 250 mcgs (Ovidrel®, MD Serono, Rockland, MA, USA). One mature oocyte was obtained at vaginal oocyte retrieval (VOR) and fertilized by intracytoplasmic sperm injection (ICSI), but the embryo did not develop into a blastocyst of sufficient quality for blastomere biopsy and cryopreservation.

During her second cycle that began 16 weeks after TLH, she received 450 units of FSH and 30 units of low dose hCG daily. Serial transvaginal ultrasound scans for monitoring were performed as before. Cetrorelix was started on day 5, and on day 9 peak estradiol was 1048 pg/mL and 3 follicles greater than 11 mm were seen (the largest measuring 22.8 mm); she was again triggered with subcutaneous Ovidrel, 250 mcgs. Two mature oocytes were retrieved at VOR and fertilized by ICSI, but no good quality blastocyst developed for biopsy or cryopreservation.

During the second VOR, no anatomic abnormalities were noted in the vagina upon initial speculum placement. Transvaginal ultrasound-guided oocyte retrieval was performed with a 17-gauge Wallace needle, and the procedure was challenging due to the deep location of ovaries and paucity of follicles. Mid procedure, a moderate amount of serosanguinous fluid was noted to be leaking from the vagina around the ultrasound probe. Following oocyte collection and removal of the ultrasound probe, speculum exam revealed an open vaginal cuff with intraperitoneal organs visible through the open cuff. There was no evidence of bowel herniation or perforation after the procedure with confirmation of ovarian structure aspiration based on retrieval of 2 oocytes. After identification of the dehiscence, the patient was awakened from anesthesia and then emergently transferred to the local university hospital to undergo immediate surgical evaluation by her gynecologic oncologist. Intra-operative findings demonstrated a 5 cm cuff defect with bladder prolapsing without bowel involvement. The dehiscence was repaired transvaginally without complication: the defect was closed with five figure-of-eight 0 Vicryl interrupted sutures with additional reinforcement of apices of each fornix and the left and right fornices with single interrupted 0 Vicryl sutures. The procedure concluded with an unremarkable cystoscopy and rectal exam. The patient had an uncomplicated post-operative course. The plan was to allow 6 months for cuff healing prior to proceeding with transabdominal oocyte retrieval if the ovaries are accessible.

## Discussion & Conclusions

While there has been a surge of publications regarding VCD in the last 10 to 15 years, much still remains to be learned. Given the rarity of the complication, it is challenging to design prospective studies or randomized controlled trials to better assess the specific risk factors that lead to VCD. Much of the current data upon which clinicians rely is abstracted from case reports, case series, and retrospective chart reviews. Our case report adds to this growing body of literature on women at risk for VCD.

In this report, we discuss the second reported case of vaginal cuff dehiscence to occur after oocyte retrieval in a woman undergoing fertility preservation treatment after hysterectomy. Prior to her procedure, our patient demonstrated some known risk factors for dehiscence. These risk factors were similarly seen in the recent first documented case of vaginal cuff dehiscence during VOR as reported by Peyser et al. [5]. For both patients, dehiscence occurred following minimally invasive hysterectomies (robotic-assisted laparoscopic in the Peyser case and laparoscopic in our case). Additionally, both women underwent hysterectomy for malignant indications, that can predispose to wound healing complications [2]. In contrast, the patient described in the Peyser case was 25 years old and obese while our patient was 35 years old with a BMI of 20.5. Obesity and older age have been shown to be protective against VCD after total laparoscopic hysterectomy according to a small case control study [6]. The Peyser patient underwent retrieval 4 months after hysterectomy, while our patient underwent VOR 12 weeks and again at 17 weeks after hysterectomy, at which time VCD occurred. In both cases the vaginal dehiscence was diagnosed intraoperatively at time of retrieval and was able to be repaired vaginally. Finally, both women were undergoing fertility treatment that likely contributed additional risk from serial transvaginal ultrasound monitoring and transvaginal egg retrievals. Fortunately, in both cases, each patient ultimately did well. Our patient was managed appropriately with immediate identification of VCD and emergent transfer to the local hospital for prompt surgical repair, as is the standard of care for VCD.

Uterine cancer is the fourth most commonly diagnosed malignancy in the US and the sixth most commonly diagnosed malignancy worldwide [7, 8]. It is one of the few cancers with increasing incidence over time. Globally, rates of uterine cancer incidence are on the rise with suspected close relationship to the ongoing obesity epidemic [8]. Within the US, this increased incidence is noted across age ranges, with increased rates of diagnosis in both pre- and post-menopausal women. Standard therapy for women with uterine cancer includes total hysterectomy, bilateral salpingo-oophorectomy and staging, followed by chemotherapy and radiation for high-risk disease or more advanced stages. For select women with lower-risk disease, fertility sparing treatment may be considered. This includes women with endometrial intraepithelial neoplasia (EIN) and grade 1 endometrioid endometrial cancer (EEC) with presumed early stage I disease who are otherwise good candidates for surgery [9]. Given that fertility sparing treatment is not standard of care, providers should expect that hysterectomy will remain the most common therapeutic pathway for women with uterine cancer.

Concurrently, women are gaining increased access to assisted reproductive technology (ART) for treatment of infertility [10]. As a result, the use of ART in fertility preservation through embryo and oocyte cryopreservation continues to rise among premenopausal women who face gynecologic malignancy through personal diagnosis or elevated genetic risk [11]. As ART allows more women to preserve their fertility and as gynecologic cancer rates continue to rise, providers will increasingly encounter women status post hysterectomy who desire continued family building via gestational carrier. Therefore, it is imperative that Ob/Gyn generalists, gynecologic oncologists, and infertility specialists be aware of the unique risks that maybe be encountered by these patients.

Taken together, these two case reports point to what could ultimately become a more common phenomenon: post-hysterectomy vaginal dehiscence after oocyte retrieval. The incidence of VCD in these cases is unknown and will likely remain rare. Currently there are no guidelines in place for women who have undergone hysterectomy with regard to when they can begin fertility treatment in the post-operative period. It may be prudent to minimize repetitive transvaginal ultrasound scanning during the IVF cycle or to extend the post-hysterectomy delay in oocyte retrieval to a minimum of 4 months in these patients (as per the timelines seen between both cases). This is much longer than the usual recommendation to avoid vaginal intercourse for 6–8 weeks after hysterectomy, but serial transvaginal procedures using an ultrasound probe associated with IVF treatment may exert focused mechanical forces on the vaginal cuff that exceed those experienced during sexual intercourse. If ovaries are accessible, transabdominal oocyte retrieval may be an option. Given both the rising incidence of women undergoing hysterectomy for gynecologic malignancies and of women accessing ART, we expect that more cases such as was seen with our patient will be experienced by other IVF practices.

## Data Availability

Not applicable.

## Abbreviations

ART: Assisted reproductive technology
EEC: Endometrioid endometrial cancer
EIN: Endometrial intraepithelial neoplasia
FSH: Follicle stimulating hormone
ICSI: Intracytoplasmic sperm injection
IUD: Intrauterine device
IVF: In-vitro fertilization
TLH: Total laparoscopic hysterectomy
VCD: Vaginal cuff dehiscence
VOR: Vaginal oocyte retrieval
